# Mediterranean Spur-Thighed Tortoises (*Testudo graeca*) Have Optimal Speeds at Which They Can Minimise the Metabolic Cost of Transport, on a Treadmill

**DOI:** 10.3390/biology11071052

**Published:** 2022-07-13

**Authors:** Heather Ewart, Peter Tickle, Robert Nudds, William Sellers, Dane Crossley, Jonathan Codd

**Affiliations:** 1 School of Biological Sciences, University of Manchester, Manchester M13 9PL, UK; heather.ewart@postgrad.manchester.ac.uk (H.E.); robert.nudds@manchester.ac.uk (R.N.); 2 School of Biomedical Sciences, University of Leeds, Leeds LS2 9JT, UK; p.tickle@leeds.ac.uk; 3 School of Natural Sciences, University of Manchester, Manchester M13 9PL, UK; william.sellers@manchester.ac.uk; 4 Department of Biological Sciences, University of North Texas, Denton, TX 76203-5017, USA; dane.crossley@unt.edu

**Keywords:** locomotion, respirometry, Testudines, shell, biomechanics, kinematics

## Abstract

**Simple Summary:**

Understanding the energy that animals use to move around is important, as it can shed light on how they make decisions about where and how to locomote. Tortoises are unique among vertebrates in having a shell, which influences almost all aspects of their biology. Here, we experimentally quantified the metabolic cost of transport in Mediterranean spur-thighed tortoises walking on a treadmill while also quantifying the kinematics of their movement. We found, in line with previous studies, that tortoises move more efficiently than predicted and present the first data demonstrating a curvilinear cost of transport over their speed range. We conclude that tortoises have an optimum speed at which they move to minimise their metabolic cost of locomotion.

**Abstract:**

Tortoises are famed for their slow locomotion, which is in part related to their herbivorous diet and the constraints imposed by their protective shells. For most animals, the metabolic cost of transport (CoT) is close to the value predicted for their body mass. Testudines appear to be an exception to this rule, as previous studies indicate that, for their body mass, they are economical walkers. The metabolic efficiency of their terrestrial locomotion is explainable by their walking gait biomechanics and the specialisation of their limb muscle physiology, which embodies a predominance of energy-efficient slow-twitch type I muscle fibres. However, there are only two published experimental reports of the energetics of locomotion in tortoises, and these data show high variability. Here, Mediterranean spur-thighed tortoises (*Testudo graeca*) were trained to walk on a treadmill. Open-flow respirometry and high-speed filming were simultaneously used to measure the metabolic cost of transport and to quantify limb kinematics, respectively. Our data support the low cost of transport previously reported and demonstrate a novel curvilinear relationship to speed in Testudines, suggesting tortoises have an energetically optimal speed range over which they can move in order to minimise the metabolic cost of transport.

## 1. Introduction

Turtles belong to the amniote clade (which also includes mammals, birds, crocodilians and lepidosaurs) and are one of the most iconic and ancient reptile groups, appearing in the fossil record over 220 million years ago. Although they are often considered “primitive”, turtles have perhaps the most derived morphology found among living amniotes: their bony shell [1]. The evolutionary origin of the shell was unclear until the discovery of *Odontochelys semitestacea* [2] and subsequent reinterpretation of *Eunotosaurus africanus* [3], which allowed the reconstruction of the character acquisitions of the modern turtle shell. The turtle shell is intriguing, as it does not originate from the skin (as is found in other bony coverings in lizards, ankylosaur and armadillos), but rather forms from integration of the ribs and vertebrae [3]. Turtles’ shells are their defining characteristic that distinguish them from all other vertebrates, encompassing an inflexible vertebral column (excluding the neck and tail), as it is fused to the carapace [4]. These adaptations to life inside a box influence three fundamental aspects of turtle biology: constraints on the way they breathe, how they reproduce and, fundamentally, how they move. 

How and why an animal moves and its interactions with the environment have a considerable influence on its daily energy budget [5]. The morphology of the structures involved in locomotion and the patterns of their movements have evolved in ways that should act to improve fitness. For many animals, the ability to increase speed is one such fitness-related trait that is paramount in their ability to avoid predators or in being a more effective, faster-moving predator themselves [6]. However, speed is not important for all animals. Tortoises, for example, are herbivores that do not need to move faster to improve their ability to catch prey, and the evolution of their shells [3] has allowed sufficient protection that, in these animals, eliminates the need for increases in speed to escape predation [6]. Tortoises are famously slow, and moving slowly with a shell does pose some unique challenges during locomotion. In almost all tetrapods, for example, a flexible vertebral column is an important contributor during locomotion. However, turtle locomotion is dependent entirely on movement of their limbs [4]. Turtles employ a sprawling gait and movements of the limb that are similar during swimming and walking [4]. This similarity in limb movement caused by their rigid bodies means that it is only the limbs that can generate propulsion. Driven by the physical constraints of their shells, tortoises use only one walking gait during terrestrial locomotion [7]. When tortoises move on land, they also abandon a stable equilibrium during their stride, meaning they are able to avoid abrupt changes in force that would reduce the efficiency of their slow muscles [8]. Testudines, therefore, accept a degree of pitching and rolling during their terrestrial movements whilst restricting fast and energetically expensive movements of the limbs and body [7]. The optimisation of a single slow-moving walking gait enables the specialisation of slow-twitch type I muscle fibres, which reduce the speed of muscle contraction (in vitro) in tortoises [9]. The rate of muscle contraction is inversely related to muscular energetic efficiency, and the predominance of slow-twitch type I muscle fibres in tortoises is thought to underpin their relatively low metabolic cost of transport (CoT) relative to other reptiles and vertebrates [10,11]. These adaptations for efficiency in terrestrial locomotion in tortoises, however, do appear to come with a trade-off should their behaviour and movement require rapid limb movements. For example, the relatively high metabolic cost of self-righting, which is up to five-fold higher than that during walking, is thought to be linked in part to the associated rapid movements of the limbs during this behaviour [12]. A slow rate of muscle contraction may therefore be adaptive for walking but not other behaviours [12].

The CoT is a useful measure for quantifying the energetic cost of transporting a unit of body weight over a given unit of distance [13]. Numerous studies have compared the CoT in a wide range of animals during terrestrial locomotion to tease apart the interaction between body size and efficiency of locomotion [14]. CoT is important for understanding route choice and movement behaviour in the context of an energetic landscape across different species [15]. The prevailing view is that energy landscapes influence fundamental aspects of movement, and data supporting this exist for fish, mammals and birds [16,17,18,19,20,21]. Understanding the metabolic cost of locomotion can ultimately shed light on an animals’ energetic budgets. Energy budgets consist of five basic components: basal metabolic rate, the costs of thermoregulation, processing food, reproductive output and locomotion. The cost of locomotion can be substantial, and insight into how these costs might change with speed is important, as an animal must maintain an overall positive energy balance (energy output ≤ energy input). Animals can minimise the energy they use to move in a number of ways, including altering the frequency of when they move or moving at their most energetically optimal speeds. Currently, there is a lack of information on perturbations in the CoT, and recent interest has begun to focus on extrapolating laboratory-based studies of locomotion to animals moving in the wild in order to determine how valid these data are for free-ranging animals [22]. Treadmill-based research can identify preferred speeds that minimise energetic cost and is important in relation to predicting how animals move in the wild. However, CoT and movement data are sparse in Testudines. For example, there are only two published accounts of the CoT in a terrestrial (Cryptodira: *Terrapene ornata* [11]) and an aquatic (Pleurodira: *Emdura macquarii* [10]) species. Both of these studies demonstrated that the CoT during terrestrial locomotion (5.97 J kg^−1^ m^−1^; 8.0 J kg^−1^ m^−1^) was around half that predicted from multi-species scaling analyses (12.55 J kg^−1^ m^−1^; 15.9 J kg^−1^ m^−1^) ([10]; [11], respectively). 

Testudines therefore present a novel opportunity to examine CoT and associated energy-saving adaptations in the context of constrained body morphology that poses unique limitations on locomotion. While turtles appear to be an outlier in expected CoT, further data are needed to confirm whether this pattern is applicable across a wider selection of Testudines. We therefore measured the CoT across a range of speeds in the Mediterranean spur-thighed tortoise (*Testudo graeca*) using respirometry. Using video analysis and respirometry, we examined the kinematics of movement and tested the hypothesis that *T. graeca* have a preferred speed for minimising the energetic cost of locomotion, on a treadmill.

## 2. Materials and Methods

### 2.1. Study Species and Husbandry

Captive-born, 2-year-old female Mediterranean spur-thighed tortoises (*T. graeca*, *N* = 5) were housed indoors within the University of Manchester’s Biological Services Facility. Tortoises were maintained under a thermal gradient from 25 °C to 35 °C [23], established using ceramic heating lamps at opposite ends of the vivarium. UV and natural light were also provided. A vivarium (2 × 0.9 × 0.7 m) was lined with fine-grain sand (to a depth of ~10 cm), and the environment was enriched by the addition of rocks, wooden logs, plants and tunnels. Tortoises were fed a mix of commercial tortoise pellets (Komodo Complete Holistic Tortoise Diet, Happy Pets Products, Leicester, UK), fresh food (vegetables, dark leafy greens, and fruit) and cuttlefish bones. The same proportion of each different food type was offered to the tortoises each day, and there was no evidence of preference for any particular food type. Individuals were not fasted prior to data collection, as tortoise digestion can span several weeks to months [24]. Food and water were available *ad libitum*. 

### 2.2. Experimental Protocol

The temperature-dependence of the physiology of ectotherms is an important factor when examining their performance, and optimal reptilian locomotor performance occurs (on average) at 35 °C [25]. Tortoises were therefore restricted to the tank hot zone for 30 min prior to trials to ensure an optimal body temperature (T_b_, 34.6 ± 0.4 °C) throughout all trials. Tortoises were trained for a relatively brief amount of time—four months—to walk on a small animal treadmill (Model: LE8710, Panlab, Harvard Apparatus, Barcelona, Spain) inside a Perspex^®^ box (20 × 15 × 10 cm). Training indicated that tortoises were restricted to a walking gait over a limited speed range (*U*, 1.2–6.0 m min^−1^); therefore, these speeds were selected as the range to be used for all experimental manipulations. During walking experiments, a randomly selected tortoise was placed into the respirometry box and resting metabolic rate (RMR) was recorded. The tortoise then walked at a randomly selected speed until a steady (~60 s) trace was achieved (3–5 min), after which post-exercise RMR was recorded. Tortoises were given at least one rest day between trials. During each trial, oxygen consumption and carbon dioxide production were measured using respirometry. A subset of these data for tortoises moving at a single speed were previously reported for comparison with the metabolic cost of self-righting [12]. These experiments were approved by the University of Manchester Animals Ethics Committee (Permit D.039) in accordance with the Animal (Scientific Procedures) Act 1986.

### 2.3. Respirometry

An open-flow respirometry system (all equipment and software: Sable Systems International^®^, Las Vegas, NV, USA) was used to measure oxygen consumption (${\dot{V}}_{O2}$) and carbon dioxide production (${\dot{V}}_{\mathrm{CO}2}$). The respirometry setup was calibrated using a known flow rate of nitrogen and found to be accurate between ±3–5%. A mass flow pump (MFS-2) was used to pull air through the respirometry chamber at 0.3 L min^−1^. Water vapour pressure (WVP) of excurrent air was measured (RH300 water vapour meter) and then scrubbed from the airstream using calcium chloride (2–6 mm granular, Merck, Germany), after which CO_2_ content was measured (CA-10a CO_2_ analyser). CO_2_ was then scrubbed from the airstream using soda lime (2–5 mm granular, Sigma-Aldrich, Darmstadt, Germany), and finally, O_2_ was measured (Oxzilla II absolute and differential dual channel O_2_ analyser). The primary flow rate (FR) was adjusted to a dry-corrected flow rate (FRc) using the standard equations in ExpeData^®^ [26]; FRc = (FR (BP-WVP))/BP, where BP is the barometric pressure. Standard equations for ${\dot{V}}_{O2}$ and ${\dot{V}}_{\mathrm{CO}2}$ [23]:

$${\dot{V}}_{O2}=\frac{{\mathrm{FR}}_{c}•\;(F_{i}O_{2}-{F_{e}}^{\prime \prime }O_{2})}{1-F_{i}O_{2}}$$

where *F*_i_O_2_ is the concentration of O_2_ flowing into the respirometry chamber and *F*_e_″O_2_ is that measured in CO_2_- and H_2_O-free air by the O_2_ analyser after leaving the chamber.

$${\dot{V}}_{CO_{2}}=\frac{{\mathrm{FR}}_{c}•\;(F_{e}^{\prime }{\mathrm{CO}}_{2}-F_{i}{\mathrm{CO}}_{2})-F_{i}{\mathrm{CO}}_{2}•{\dot{V}}_{O_{2}}}{1-F_{i}{\mathrm{CO}}_{2}}$$

where *F*_i_CO_2_ is the concentration of CO_2_ in air entering the respirometry chamber and *F*_e_′CO_2_ is excurrent CO_2_ measured in H_2_O-free air by the CO_2_ analyser after leaving the chamber. These values were then used to calculate the respiratory exchange ratio (RER = ${\dot{V}}_{\mathrm{CO}2}$:${\dot{V}}_{O2}$), which informed calculation of the mass-specific power consumption (P_met_, W kg^−1^) via adjustment of ${\dot{V}}_{O2}$ by the calorific equivalent (Joules) taken from (Table 12.1 [27]) (P_met_ = ${\dot{V}}_{O2}$ × RER calorific equivalent × 4.184). The metabolic cost of transport (CoT, J kg^−1^ m^−1^) was calculated by dividing P_met_ by speed (*U*, m min^−1^). Mass-specific minimum CoT_min_, (J kg^−1^ m^−1^) was calculated by taking the average of the minimum CoT of each individual from each trial.

### 2.4. Kinematics

A Sony^®^ cyber-shot camera (DSC-RX-10 III, Sony Corporation^®^, Tokyo, Japan) was used to film all trials at 100 frames per second. The kinematics of locomotion—duty factor (DF), stride length (*l*_stride_, m), stride frequency (*f*_stride_, Hz) and the duration of both stance phase (*t*_stance_, s) and swing phase (*t*_swing_, s) at each walking speed (*U*, m min^−1^)—were determined using Tracker v.2.51 (https://physlets.org/tracker, The Open Source Physics Project, Aptos, California, USA, accessed on 25 April 2021). The forelimb nearest the camera was tracked for kinematic analyses.

### 2.5. Statistical Analyses

All statistical analyses (Table 1) were performed using R statistical software (version 3.6.2, R Core Team 2020, http://www.R-project.org/, Vienna, Austria, accessed on 15 June 2021). The Akaike Information Criterion (AIC) was used to determine the line of best fit for the relationship between CoT and *U*. The effects of *U* on the kinematic parameters (*l*_stride_, *f*_stride_, *t*_stance_, *t*_swing_, DF) and energetics (${\dot{V}}_{O2}$, ${\dot{V}}_{\mathrm{CO}2}$, P_met_, RER) were tested using regression. All analyses (except mass-specific P_met_ and CoT) included mass as a proxy for individual effects to test individual effects on each dependent variable, as well as to consider any interactions between mass and *U*. In cases with no interaction between the independent variables, mass was removed from the test and the regression was conducted again with only *U* and the dependent variable. Statistical differences were considered significant when *p* < 0.05 and r^2^ values are adjusted. All data mean values are displayed ± standard error (SE). Processed summary energetics and kinematics datasets can be found in the electronic Supplementary Materials.

## 3. Results

### Energetics and Kinematics of Locomotion

**Figure 1:**
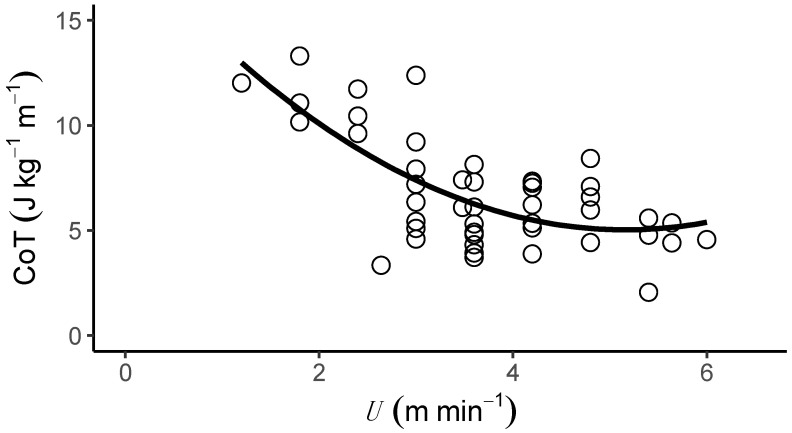
The metabolic cost of transport (CoT, J kg^−1^ m^−1^) of locomotion on a level treadmill over a range of speeds (*U*, m min^−1^) in the Mediterranean spur-thighed tortoise (*T. graeca*). The relationship between CoT and *U* is best described by a curvilinear fit (Table 1), which suggests that Mediterranean spur-thighed tortoises have an optimal speed (between 4–6 m min^−1^) at which the CoT is minimised.

**Figure 2:**
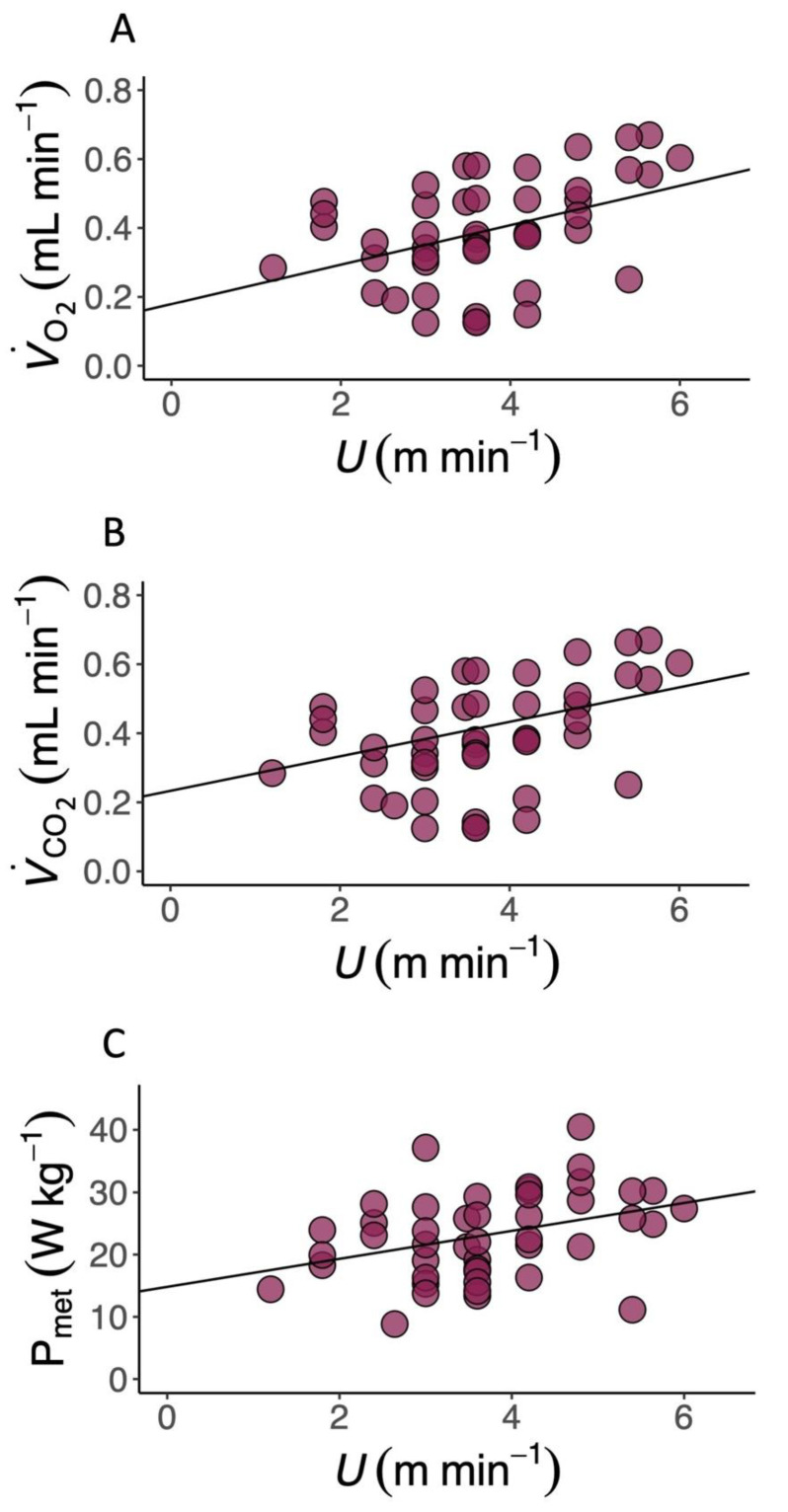
Locomotor energetics in the Mediterranean spur-thighed tortoise (*T. graeca*). (**A**) The rate of oxygen consumption (${\dot{V}}_{O2}$, mL min^−1^), (**B**) the rate of carbon dioxide production (${\dot{V}}_{\mathrm{CO}2}$, mL min^−1^) and (**C**) mass-specific metabolic power (P_met,_ W kg^−1^) during locomotion on a level treadmill over a range of speeds (*U*, m min^−1^) in the Mediterranean spur-thighed tortoise (*T.graeca*). ${\dot{V}}_{O2}$, ${\dot{V}}_{\mathrm{CO}2}$, and P_met_ all increase linearly with *U* (Table 1).

**Figure 3:**
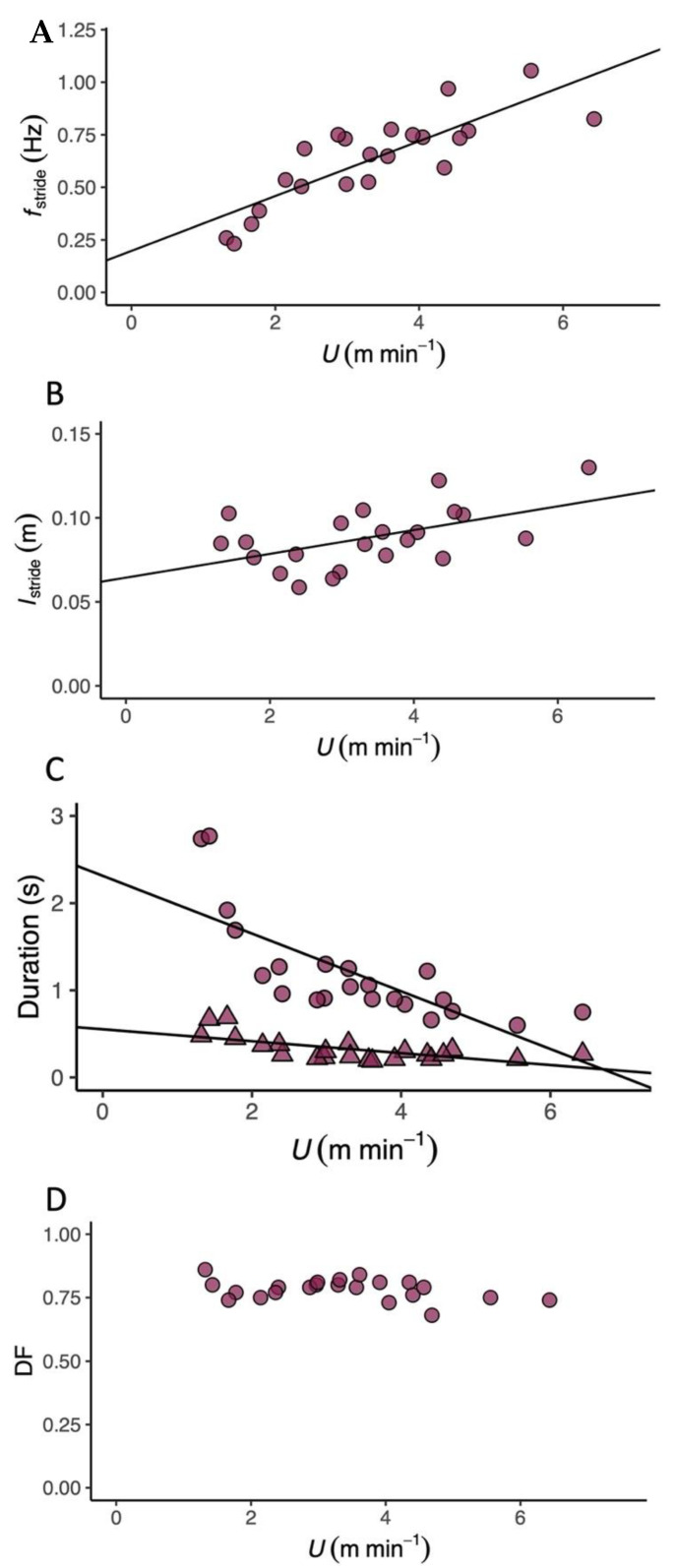
Locomotor kinematics in the Mediterranean spur-thighed tortoise (*T.graeca*). (**A**) Stride frequency (*f_s_*_tride_, Hz, circles) increased linearly with increasing speed (*U*, m min^−1^). (**B**) Stride length (*l*_stride_, m, circles) also increased linearly with increasing *U*. (**C**) Swing (*t*_swing_, triangles) and stance (*t*_stance_, circles) durations (s) both decreased linearly with increasing U. *t*_stance_ was always greater than *t*_swing_. (**D**) Duty factor (DF, circles) was not affected by *U*. The lines of best fit and associated statistical output are presented in Table 1.

*T. graeca* walked over a very slow speed range (*U*, 1.2–6.0 m min^−1^). The metabolic cost of transport (CoT) differed predictably with *U* (Table 1 and Table S1), and the relationship between CoT and *U* was best described by a second-order polynomial (Figure 1 and Figure S1, 2nd order polynomial: AIC = 192.99, Delta AIC = 0.00 and linear regression: AIC = 197.87, Delta AIC = 4.88). The average CoT across all U was 6.93 (J kg^−1^ m^−1^), and there was an optimal speed range of 4–6 m min^−1^ at which the CoT could be minimised. ${\dot{V}}_{O2}$ (Figure 2A, Table 1, Table S1), ${\dot{V}}_{\mathrm{CO}2}$ (Figure 2B, Table 1) and mass-specific metabolic power consumption (Figure 2C) increased with increasing *U* (Table 1). *f*_stride_ (Figure 3A) and *l*_stride_ (Figure 3B) both increased with increasing *U* and mass (Table 1, Table S2 and S3). The duration of *t*_stance_ and *t*_swing_ (Figure 3C) also increased with increasing U and mass (Table 1, Table S2 and S4–S12). DF (Figure 3D, 0.78 ± 0.01) was not detectably affected by *U* (Table 1 and Table S2) and was within the range expected for a slow walking gait [28]. The distribution of selected speeds (Figure S2) indicated a preference for mid-range speeds. The overall mean value of the RER was 1.0 ± 0.02 but it was not associated with mass (Table 1, Table S1, S2 and S4–S12).

## 4. Discussion

Gaining insight from laboratory-based studies that measure the metabolic cost of locomotion is important, as these data, collected under controlled conditions, can provide a foundation for future field research where hypotheses on energy use in the wild can be examined. Our data confirm that the metabolic cost of transport (CoT) in Testudines is lower than expected when compared to other amniotes [29,30]. Additionally, we present the first evidence of a curvilinear relationship between the CoT and speed in a tortoise, suggesting that Testudines, in line with animals as diverse as horses [31], camels and donkeys [32], humans [33,34], emus and ostriches [35], barnacle geese [36] and the Svalbard rock ptarmigan [37], have a speed range over which the CoT can be minimised. Understanding the significance of how animals choose to move is important, as the consequences of these decisions form part of the process for balancing their energy budget in a heterogeneous landscape. Freely-moving animals are expected to minimise CoT by travelling at less energetically expensive speeds [15] and by selecting routes that limit unnecessary increases in CoT [19]. These expectations are largely thought to be consistent with natural selection due to the supposed advantages for overall fitness in reducing the energy expenditure for any given task. Specifically, in terms of their locomotion, animals can also select speed and gait in response to different factors (i.e., gradients, substrates, environmental conditions, the requirements of specific activities, such as foraging), and feedback from each of these can dictate movement choices and ultimately energetic cost [22,38]. Although the ability to optimise energetic efficiency can have substantial impacts on free-ranging animals, the majority of research into the energetics and biomechanics of terrestrial locomotion has tended to focus on experimental manipulations on a treadmill, as with the research presented here. For many terrestrial species, even data such as speed and gait classifications have yet to be addressed in animals moving under natural conditions [22]. Locomotion data are particularly poor for Testudines (currently these data exist for only 3 out of over 250 species), which may be due in part to their reluctance to walk under laboratory conditions and the time required to successfully train these animals to move on treadmills. 

Datasets such as the one collected here provide a unique opportunity to address the interplay between laboratory and field research. Our data would suggest that future research placing inferences into speed selection and the subsequent energetic consequences from treadmill-based studies into context for wild tortoises would be beneficial. Attempts have been made to examine the habitat–species relationship of *T. graeca* in relation to climate, relief, lithography and land use [39]. For example, it has been documented that *T. graeca* prefer open shrubland and cropland habitats over more complex mixed shrublands in the wild [39]. However, data on any potential effects of habitat on how these animals move within a given environment are lacking. Our results suggest that future work should test the hypothesis that tortoises may be able to move more often in their faster, more economical speed range in these open areas. Furthermore, this research offers a potential explanation for them preferentially selecting these habitats and ultimately provides insight into the links between habitat selection and energetic landscapes in this species. 

The CoT in Testudines has only previously been examined using respirometry twice [10,11]. In order to compare the minimum cost in these studies, the CoT_min_ for *T. graeca* was calculated by averaging the minimum CoT in each individual. In the current study, we recorded a CoT_min_ (J kg^−1^ m^−1^) of 4.11, compared to 5.97 [10] and 8.0 [11] reported for other species. Our results indicate that the tortoises in the current study have a broadly comparable minimum CoT, but it is still the lowest reported in Testudines. While interspecies variation likely explains most of the differences in CoT between studies, there are also minor methodological variations in how data were collected in previous studies that should be considered. For example, O_2_ measurements are most accurate when taken from air that is first scrubbed of water vapour and CO_2_, as in the current study (see Methods). However, the respirometry setup of previous experiments achieved this via different experimental protocols [10,11], which used a column of Drierite^®^ to dry air before it was passed through to measure oxygen concentrations. Drierite^®^ is known to have an affinity for CO_2_ and has an adverse effect on the washout characteristics of this gas, which ultimately skews the measurement of CO_2_ [40]. Furthermore, this effect is sensitive to a variety of factors (i.e., exposure time of Drierite^®^ to room air, frequency of recharge, ambient temperature, relative humidity, and state of activity being measured) [40], making it difficult to quantify the level at which it may have impacted O_2_ concentrations in previous experiments [10,11]. The use of this drying chemical is also a particular problem when recording non-steady-state measurements [40], as is the case with turtles. 

The low CoT in tortoises is in part a product of the predominance of slow-twitch type I fibres in their limb muscles, which contract at a slow, highly energetically efficient rate [9]. The evolution of a protective carapace in tortoises eliminated their need for quick escape from predators, driving the optimisation of one slow-moving gait. However, our study presents an interesting and novel finding—despite the evolution of a single very specialised, slow-moving gait functioning within a limited speed range, tortoises still demonstrate a curvilinear relationship of CoT and speed. At first glance, these animals appear to be limited in their locomotor ability; however, this is a misleading preconception. Although they have a narrow overall speed range, they are still able to locomote at optimal speeds over which the CoT can be minimised. The identification of optimal speeds is in line with predictions based on the importance of reducing energetic output for an animal’s movement ecology. Understanding the CoT and selection of preferred speeds can also shed light on how energetic constraints within an animal’s natural environment influence movement behaviour and route choice. Ultimately, based on the research presented here, tortoises would be predicted to select speeds within their preferred speed range that minimise the CoT relative to variation in their energetic landscape. 

The mean duty factor of tortoises in this study (0.78) is consistent with the typical gait theoretically predicted for tortoises should they adopt a stable gait across their speed range [8,29]. It is clear that spur-thighed tortoises are only capable of moving with a single, specialised gait (walking at low speeds). This optimised gait, which restricts unwanted displacements of body equilibrium, in conjunction with the evolution of slow-twitch muscles, functions to minimise metabolic energy costs during locomotion [8,26]. The high duty factor in tortoises can be attributed to this use of only one very slow gait, during which stance phase takes up the majority of time during a stride relative to swing phase [28]. In fact, Schmidt et al. [41] suggest that long durations of the tripedal stance phase coupled with powerful acceleration during the short bipedal swing phase are the result of the economic force of the extremely slow and efficient muscles of tortoises and are also likely a product of slow muscle contraction [9,29]. Of the 11 reptilian, three amphibian, and three mammalian species classified as “slow-moving animals” [9,29], the common link between groups is slow movement and slow muscle contraction related to high duty factor (>0.80), which is likely a strategy to limit muscle activation costs [28]. The kinematic constraints of slow movement in tortoises are unique, however, as the animals must maintain equilibrium during their gait with the added anatomical constraint of a rigid, inflexible dorsal carapace. Compared to other slow-moving animals, the evolution of a rigid carapace limits basic aspects of movement [8]. These include lateral bending of the dorsum, which further limits extension of the limbs, and variability in footfall sequencing, the latter of which is a primary mechanism in reducing the energetic expenditure of locomotion by allowing for the maintenance of dynamic stability of the body during locomotion [8,42]. 

Tortoises therefore must minimise unwanted displacements in equilibrium during their gait, which become more significant at greater stride durations (such as those seen in slow-moving animals) [29,43]. In animals that regularly locomote at duty factors greater than 0.75, maintaining a relatively fixed centre of mass is crucial for limiting displacements from body equilibrium [8,29]. However, tortoises are subject to the anatomical restraints of a low-slung trunk and limited limb extension, which influence the animals’ trunks to rise and fall and/or roll laterally during a stride [8,42]. Such departures from equilibrium not only require energy to return back to equilibrium, but also increase the likelihood of tortoises bumping the plastron against the ground. Indeed, this behaviour was observed in *T. graeca* of the present study at lower speeds; animals were more likely to stray from steady walking in the centre of the treadmill and/or bump their plastron against the treadmill at unsustainably slow speeds (which may be related to the increased CoT at low speeds). Fast-twitch muscle fibres can be utilised by other groups of amniotes to maintain centre of mass [44]. However, the specialised mechanics of the tortoise gait allows for the minimisation of body displacement during stride without the requirement of fast-twitch muscle fibres, facilitating the evolution and energetic optimisation of slow-twitch muscle fibres [8,9,29]. The low metabolic cost of locomotion in tortoises here supports previous findings that the specialisation of slow-twitch muscle fibres, coupled with the optimisation of the tortoise gait, are key drivers in the observed curvilinear relationship between speed and CoT. As speed increases, unwanted displacements become easier to control within the animals’ preferred speed range, lowering the metabolic cost of moving.

## 5. Conclusions

The Testudines carapace may constrain range of movement, but it is clear that tortoises possess unique adaptations that minimise locomotor energetics despite these anatomical constraints. Our data confirm links made between the specialisation of tortoise gait and the energetic efficiency of their locomotion, while providing novel insight into the curvilinear relationship between speed and CoT in tortoises. This relationship indicates that tortoises have a range of speeds that minimise the metabolic cost of moving. Relating CoT to speed selection under a laboratory setting provides data that can be used to predict and better understand movement within an energetic landscape in free-ranging tortoises. We would suggest that future research on preferred speed ranges in wild *T. graeca* and other Testudines would be beneficial to test hypotheses on the optimisation of their energetic balance by minimising CoT within an energetic landscape. 

## Figures and Tables

**Table 1:** The results of statistical tests conducted to determine the effects of speed (*U*) and mass on the energetic and kinematic variables. No interactions between mass and *U* were found for any of the dependent variables, so the statistical models were reduced to main effect terms only.

Dependent Variable	Equation Describing Line of Best Fit	Independent Variable	
		Speed (*U*)	Body Mass
Energetics			
CoT	*y* = −5.23x^2^ + 0.51*x* + 18.533	*F*_2,42_ = 19.89 r^2^ = 0.462 *p* < 0.001	
${\dot{V}}_{O2}$	*y* = 0.06*x* + 0.179	*F*_1,43_ = 9.41 r^2^ = 0.160 *p* = 0.004	*F*_1,42_ = 37.05 r^2^ = 0.412 *p* < 0.001
${\dot{V}}_{\mathrm{CO}2}$	*y* = 0.05*x* + 0.233	*F*_1,43_ = 5.95 r^2^ = 0.101 *p* = 0.019	*F*_1,42_ = 39.38 r^2^ = 0.450 *p* < 0.001
P_met_	*y* = 2.25*x* + 14.81	*F*_1,43_ = 6.58 r^2^ = 0.113 *p* = 0.014	
RER	*y* = −0.02*x* + 1.154	*F*_1,43_ = 1.78 r^2^ = 0.017 *p* = 0.190	*F*_1,42_ = 1.15 r^2^ = 0.025 *p* = 0.290
Kinematics			
*f*_stride_	*y* = 0.13*x* + 0.198	*F*_1,20_ = 43.12 r^2^ = 0.667 *p* < 0.001	*F*_1,19_ = 31.60 r^2^ = 0.195 *p* < 0.001
*l*_stride_	*y* = 0.007*x* + 0.064	*F*_1,20_ = 7.70 r^2^ = 0.242 *p* = 0.012	*F*_1,19_ = 29.43 r^2^ = 0.444 *p* < 0.001
*t*_swing_	*y* = −0.07*x* + 0.553	*F*_1,20_ = 14.65 r^2^ = 0.394 *p* = 0.001	*F*_1,19_ = 16.07 r^2^ = 0.261 *p* < 0.001
*t*_stance_	*y* = −0.33*x* + 2.314	*F*_1,20_ = 25.64 r^2^ = 0.540 *p* < 0.001	*F*_1,19_ = 10.11 r^2^ = 0.151 *p* = 0.005
DF	*y* = −0.01*x* + 0.818	*F*_1,20_ = 3.02 r^2^ = 0.088 *p* = 0.098	*F*_1,19_ = 0.90 r^2^ = 0.039 *p* = 0.355

## Data Availability

The data presented in this study are openly available in FigShare at https://figshare.com/s/577b43a3aa0568aca269, accessed on 7 July 2022.

## Supplementary Materials

The following supporting information can be downloaded at https://www.mdpi.com/article/10.3390/biology11071052/s1: raw and processed energetics (Table S1, Figure S1) and kinematics (Table S2–S12) datasets and a figure of the frequency distribution of speed selection (Figure S2).

## References

[1] Joyce W.G., Gauthier J.A. (2004). Paleoecology of Triassic stem turtles sheds new light on turtle origins. Proc Roy. Soc. B..

[2] Li C., Wu X.-C., Rieppel O., Wang L.-T., Zhao L.-J. (2008). An ancestral turtle from the Late Triassic of southwestern China. Nature.

[3] Lyson T.R., Schachner E.R., Botha-Brink J., Scheyer T.M., Lambertz M., Bever G.S., Rubidge B.S., de Queiroz K. (2014). Origin of the unique ventilatory apparatus of turtles. Nat. Commun..

[4] Gillis G.B., Blob R.W. (2001). How muscles accommodate movement in different physical environments: Aquatic vs. terrestrial locomotion in vertebrates. Comp. Biochem. Physiol..

[5] Cavagna G.A., Kaneko M. (1977). Mechanical work and efficiency in level walking and running. J. Physiol..

[6] Alexander R.M. (2003). Principles of Animal Locomotion.

[7] Alexander R.M. (1993). Gaits of Mammals and Turtles. J. Roy. Soc. Jap..

[8] Jayes A.S., Alexander R.M. (1980). The gaits of chelonians: Walking techniques for very low speeds. J. Zool..

[9] Woledge R.C. (1968). The energetics of tortoise muscle. J. Physiol..

[10] Baudinette R.V., Miller A.M., Sarre M.P. (2000). Aquatic and Terrestrial Locomotory Energetics in a Toad and a Turtle: A Search for Generalisations among Ectotherms. Physiol. Biochem. Zool..

[11] Zani P.A., Kram R. (2008). Low metabolic cost of locomotion in ornate box turtles, Terrapene ornata. J. Exp. Biol..

[12] Ewart H.E., Tickle P.G., Sellers W.I., Lambertz M., Crossley N.D.A., Codd J.R. (2022). The metabolic cost of turning right side up in the Mediterranean spur-thighed tortoise (Testudo graeca). Sci. Rep..

[13] Tucker V.A. (1970). Energetic cost of locomotion in animals. Comp. Biochem. Physiol..

[14] Nudds R.L., Codd J.R., Sellers W.I. (2009). Evidence for a mass dependent step-change in the scaling of efficiency in terrestrial locomotion. PLoS ONE.

[15] Shepard E., Wilson R., Rees W.G., Edward G., Sergio A.L., Simon B.V., Associate Editor: Robert D., Editor: Judith L.B. (2013). Energy Landscapes Shape Animal Movement Ecology. Am. Nat..

[16] Mosser A., Avgar T., Brown G., Walker C., Fryxell J. (2014). Towards an energetic landscape: Broad-scale accelerometry in woodland caribou. J. Anim. Ecol..

[17] Masello J.F., Kato A., Sommerfeld J., Mattern T., Quillfeldt P. (2017). How animals distribute themselves in space: Variable energy landscapes. Front. Zool..

[18] Wilson R., Flavio Q., Victoria J.H. (2012). Construction of energy landscapes can clarify the movement and distribution of foraging animals. Proc. R. Soc. B Biol. Sci..

[19] Wall J., Douglas-Hamilton I., Vollrath F. (2006). Elephants avoid costly mountaineering. Curr. Biol..

[20] Brownscombe J.W., Gutowsky L.F.G., Danylchuk A.J., Cooke S.J. (2014). Foraging behaviour and activity of a marine benthivorous fish estimated using tri-axial accelerometer biologgers. Mar. Ecol. Prog. Ser..

[21] Amélineau F., Fort J., Mathewson P.D., Speirs D.C., Courbin N., Perret S., Porter W.P., Wilson R.J., Grémillet D. (2018). Energyscapes and prey fields shape a North Atlantic seabird wintering hotspot under climate change. R. Soc. Open Sci..

[22] Marmol-Guijarro A.C., Nudds R.L., Marrin J.C., Folkow L.P., Codd J.R. (2019). Terrestrial locomotion of the Svalbard rock ptarmigan: Comparing field and laboratory treadmill studies. Sci. Rep..

[23] Lambert M.R.K. (1981). Temperature, activity and field sighting in the mediterranean spur-thighed or common garden tortoise Testudo graeca L. Biol. Conserv..

[24] Tracy C.R., Zimmerman L.C., Tracy C., Bradley K.D., Castle K. (2006). Rates of Food Passage in the Digestive Tract of Young Desert Tortoises: Effects of Body Size and Diet Quality. Chelonian Conserv. Biol..

[25] Lailvaux S.P. (2007). Interactive effects of sex and temperature on locomotion in reptiles. Int. Comp. Biol..

[26] Lighton J.R. (2008). Measuring Metabolic Rates: A Manual for Scientists.

[27] Brody S. (1945). Bioenergetics and Growth, with Special Reference to the Efficiency Complex in Domestic Animals.

[28] Usherwood J.R., Self Davies Z.T. (2017). Work minimization accounts for footfall phasing in slow quadrupedal gaits. eLife.

[29] Alexander R.M. (1993). Optimization of structure and movement of the legs of animals. J. Biomech..

[30] Full R.J., Gnaiger W.W.A.E. (1989). Mechanics and energetics of terrestrial locomotion: Bipeds to polypeds. Energy Transformations in Cells and Organisms.

[31] Hoyt D.F., Taylor C.R. (1981). Gait and the energetics of locomotion in horses. Nature.

[32] Maloiy G.M.O., Rugangazi B.M., Rowe M.F. (2009). Energy expenditure during level locomotion in large desert ungulates: The one-humped camel and the domestic donkey. J. Zool..

[33] Margaria R. (1938). Sulla fisiologia e specialmente sul consumo energetico della marcia e della corsa a varie velocita ed inclinazioni del terreno. Atti Accad. Naz. Lincei Memorie.

[34] Margaria R., Cerretelli P., Aghemo P., Sassi G. (1963). Energy cost of running. J. Appl. Physiol..

[35] Watson R.R., Rubenson J., Coder L., Hoyt D.F., Propert M.W.G., Marsh R.L. (2011). Gait-specific energetics contributes to economical walking and running in emus and ostriches. Proc. R. Soc. B Biol. Sci..

[36] Nudds R.L., Gardiner J.D., Tickle P.G., Codd J.R. (2010). Energetics and kinematics of walking in the barnacle goose (Branta leucopsis). Comp. Ciochem. Physiol. Part A Mol. Integr. Physiol..

[37] Nudds R.L., Folkow L.P., Lees J.J., Tickle P.G., Stokkan K.A., Codd J.R. (2011). Evidence for energy savings from aerial running in the Svalbard rock ptarmigan (Lagopus muta hyperborea). Proc. R. Soc. B Biol. Sci..

[38] Sears M.W., Riddell E.A., Rusch T.W., Angilletta M.J. (2019). The World Still Is Not Flat: Lessons Learned from Organismal Interactions with Environmental Heterogeneity in Terrestrial Environments. Int. Comp. Biol..

[39] Anadón J.D., Giménez A., Martínez M., Martínez J., Pérez I., Esteve M.A. (2006). Factors determining the distribution of the spur-thighed tortoise Testudo graeca in south-east spain: A hierarchical approach. Ecography.

[40] White C.R., Portugal S.J., Martin G.R., Butler P.J. (2006). Respirometry: Anhydrous Drierite Equilibrates with Carbon Dioxide and Increases Washout Times. Physiol. Biochem. Zool..

[41] Schmidt M., Mehlhorn M., Fischer M.S. (2016). Shoulder girdle rotation, forelimb movement and the influence of carapace shape on locomotion in Testudo hermanni (Testudinidae). J. Exp. Biol..

[42] Hildebrand M. (1977). Analysis of Asymmetrical Gaits. J. Mammal..

[43] Gray J. (1944). Studies in the mechanics of the tetrapod skeleton. J. Exp. Biol..

[44] McGhee R.B., Frank A.A. (1968). On the stability properties of quadruped creeping gaits. Math. Biosci..

